# Influence of Serum Carbamazepine Concentration on the Anticoagulant Effect of Warfarin in a Pediatric Case: Effects of Carbamazepine Tapering and Discontinuation During Concomitant Sodium Valproate Therapy

**DOI:** 10.1002/ccr3.72239

**Published:** 2026-03-12

**Authors:** Yuki Shirayama, Yu Takasao, Toshiyuki Shinozaki, Masayuki Abiko, Kota Suzuki, Kosuke Doki

**Affiliations:** ^1^ Tsuruha Inc Sapporo Japan; ^2^ Department of Pediatrics Nihonkai General Hospital Yamagata Japan; ^3^ Department of Pediatrics Kitamurayama Hospital Yamagata Japan; ^4^ Department of Pediatrics Yamagata University Hospital Yamagata Japan; ^5^ Department of Pharmaceutical Sciences, Institute of Medicine University of Tsukuba Ibaraki Japan

**Keywords:** carbamazepine, CYP2C9, PT‐INR, sodium valproate, warfarin

## Abstract

This case demonstrates an inverse relationship between serum carbamazepine concentration and the prothrombin time–international normalized ratio (PT‐INR) adjusted for the warfarin dose. The concomitant use of sodium valproate further contributes to an unstable PT‐INR, emphasizing the importance of careful and continuous monitoring of anticoagulant effects.

## 1 Introduction

Warfarin, an anticoagulant, is used as a racemic mixture of *S* and *R* optical isomers [1]. The anticoagulant effect of warfarin is influenced by changes in the pharmacokinetics of *S*‐warfarin, whose anticoagulant activity is approximately three‐ to five‐fold higher than that of *R*‐warfarin. *S*‐warfarin is metabolized primarily by cytochrome P450 (CYP) 2C9 [1, 2]. Concomitant use of CYP2C9 inducers, such as carbamazepine, increases the hepatic metabolism of *S*‐warfarin and attenuates the anticoagulant effect of warfarin [3, 4]. However, the effect of changes in serum carbamazepine concentration on the anticoagulant effect of warfarin remains unclear. Although the concomitant use of CYP2C9 inhibitors, such as sodium valproate, potentiates the anticoagulant effect of warfarin [5], there is insufficient information on the variation in the anticoagulant effect of warfarin when both carbamazepine and sodium valproate are co‐administered. This case report describes changes in the anticoagulant effect of warfarin in a pediatric patient with epilepsy and dilated cardiomyopathy after the initiation, titration, and tapering of carbamazepine, with a subsequent switch to sodium valproate and eventual discontinuation of carbamazepine.

## 2 Case History/Examination

**FIGURE 1:**
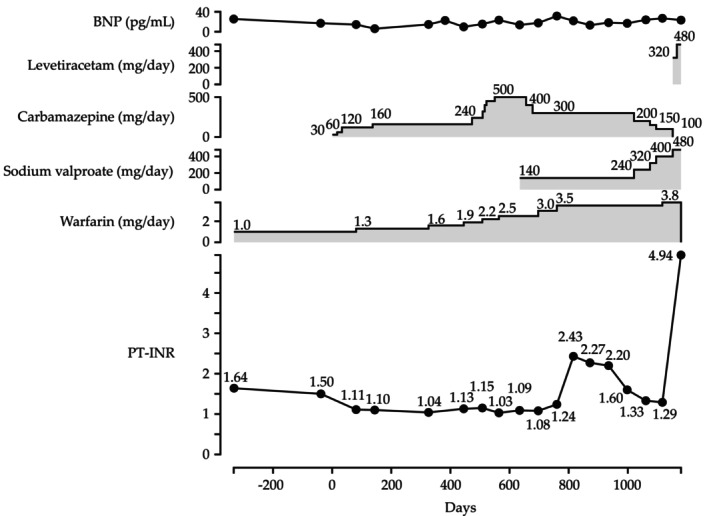
Clinical course of the present case. BNP, B‐type natriuretic peptide; PT‐INR, prothrombin time–international normalized ratio.

The patient was a 7‐year‐old boy who had been receiving warfarin for the treatment of dilated cardiomyopathy since the age of 3 months, with a PT‐INR consistently maintained within the range of 1.5–2.5. At the age of 4 years, while receiving a daily dose of 1.0 mg warfarin, he was started on carbamazepine at 30 mg/day for the management of epilepsy (on Day 1; Figure 1). The initial phase of carbamazepine induction was managed in the hospital; thereafter, treatment was continued during outpatient visits. The patient's dilated cardiomyopathy and epilepsy were treated at different hospitals; however, prescriptions from both hospitals were dispensed at our community pharmacy. Following the introduction of carbamazepine, the PT‐INR decreased to 1.11 on Day 81. Subsequently, the carbamazepine doses were maintained between 120 and 500 mg/day, and warfarin was titrated up to 3.0 mg/day. Despite these adjustments, the PT‐INR ranged from 1.03 to 1.24, which was deemed inadequate for therapeutic efficacy of warfarin (Days 144–760). On Day 640, sodium valproate was introduced at a dose of 140 mg/day to manage the patient's epilepsy. After the warfarin dose was increased to 3.5 mg/day on day 760, the PT‐INR increased to 2.43 but subsequently decreased, reaching 1.29 on Day 1117, despite no further change in the warfarin dose. Subsequently, the warfarin dose was increased to 3.8 mg/day on Day 1117. On Day 1152, carbamazepine was discontinued and levetiracetam was initiated at 320 mg/day. On Day 1180, the PT‐INR was elevated to 4.94, and warfarin was subsequently discontinued. There were no symptoms of subcutaneous bleeding on the face, body, or limbs and no bleeding from the gums. At that time, concomitant medications were soluble ferric pyrophosphate 60 mg/day, aspirin 80 mg/day, furosemide 22 mg/day, spironolactone 22 mg/day, enalapril maleate 6.4 mg/day, carvedilol 6.4 mg/day, magnesium oxide 664 mg/day. Liver and kidney functions remained within the normal ranges: aspartate aminotransferase, 21 U/L; alanine aminotransferase, 16 U/L; and serum creatinine, 0.28 mg/dL. Cardiac function was preserved, with a B‐type natriuretic peptide (BNP) level of 23.3 pg/mL and a left ventricular ejection fraction (LVEF) of 34%–40%. The patient weighed 11.5 kg at the initiation of carbamazepine, and 16.1 kg at the discontinuation of warfarin.

**FIGURE 2:**
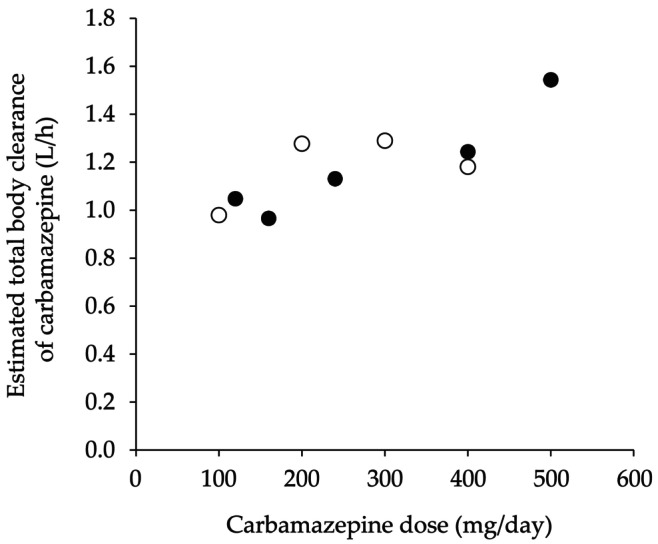
Relationship between dose and estimated total body clearance of carbamazepine. Open and closed circles represent values with and without co‐administration of sodium valproate, respectively.

**FIGURE 3:**
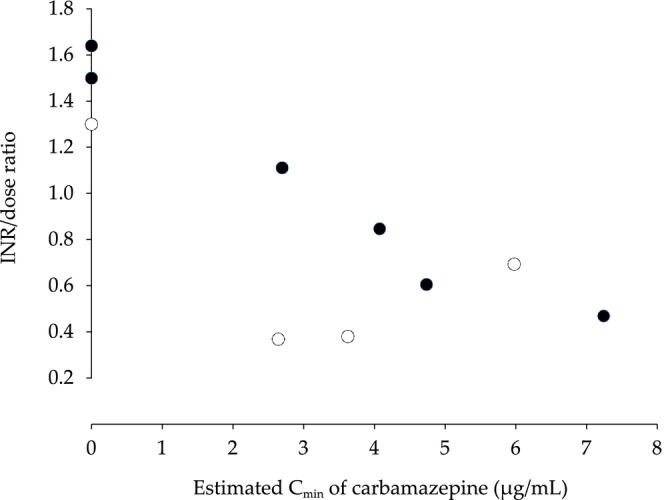
Relationship between prothrombin time‐international normalized ratio/warfarin daily dose (INR/dose) ratio and estimated minimum serum concentration (*C*_min_) of carbamazepine with and without co‐administration of sodium valproate. Open and closed circles represent values with and without co‐administration of sodium valproate, respectively.

Table 1 shows the serum concentrations and pharmacokinetic parameters of carbamazepine. The pharmacokinetic parameters were estimated using the OptjpWin Spreadsheet with Bayesian methods, based on previously reported population pharmacokinetic data from Japanese individuals [6, 7]. Figure 2 shows the relationship between the dose and estimated total body clearance (CL) of carbamazepine. Carbamazepine CL increased at higher doses regardless of the coadministration of sodium valproate. Figure 3 shows the relationship between the estimated minimum serum concentration (*C*_min_) of carbamazepine and the PT‐INR per daily warfarin dose (INR/dose ratio). The INR/dose ratio was selected for use in this study as it has been employed in previous reports to assess the effects of concomitant use with CYP2C9 inducers [3]. Before co‐administration of sodium valproate, the INR/dose ratio was negatively correlated with the estimated *C*_min_ of carbamazepine (Pearson's correlation coefficient, −0.977; *p* = 0.0003). In contrast, during co‐administration with sodium valproate, no consistent trend was observed in the relationship between carbamazepine *C*_min_ and the INR/dose ratio. When sodium valproate was co‐administered at doses of 240 and 400 mg/day, the estimated *C*_min_ of sodium valproate was 25.0 and 46.2 μg/mL, respectively, as determined using a Bayesian estimation method based on previously published Japanese population pharmacokinetic data [8].

**TABLE 1:** Serum concentration data and estimated pharmacokinetic parameters of carbamazepine.

Day after the initiation of carbamazepine	52	177	501	522	587	678	823	1050	1146
Carbamazepine dose (mg/day)	120	160	240	400	500	400	300	200	100
Time from the last dose to blood sampling (h)	15.4	15.8	16.0	7.2	15.8	8.8	14.4	5.3	6.1
Serum carbamazepine concentration (μg/mL)	2.9	4.3	4.9	9.8	5.7	9.6	5.4	6.6	4.1
Estimated CL (L/h)	1.05	0.97	1.13	1.24	1.54	1.18	1.29	1.28	0.98
Estimated *C*_min_ (μg/mL)	2.7	4.1	4.7	8.4	7.2	9.2	6.0	3.6	2.6

*Note:* Carbamazepine was administered thrice a day. Total body clearance (CL), minimum serum concentration (*C*_min_) of carbamazepine were estimated using Bayesian methods.

## 3 Discussion

In this case, we present findings suggesting that, during the co‐administration of the CYP2C9 inducer carbamazepine and the CYP2C9 inhibitor sodium valproate, changes in the anticoagulant effect of warfarin cannot be explained solely by serum carbamazepine concentrations or warfarin dosage. The anticoagulant effect of warfarin decreased with carbamazepine in a concentration‐dependent manner during upward titration. During the tapering of the carbamazepine dose during co‐administration with sodium valproate, the anticoagulant effect of warfarin was unstable and exhibited no concentration‐dependent association with carbamazepine. After carbamazepine was discontinued, the anticoagulant effect of warfarin increased markedly, as seen through the PT‐INR exceeding the therapeutic range. These findings suggest that tapering and subsequent discontinuation of carbamazepine may lead to uncontrolled PT‐INR, although the decrease in the anticoagulant effect of warfarin by carbamazepine depends on its serum concentration during upward titration.

CYP2C9‐associated drug–drug interactions between warfarin and carbamazepine have been reported in several studies [3, 9, 10]. Carbamazepine decreases the PT‐INR by 21%–61% in patients receiving anticoagulation therapy using warfarin [4, 11]. In this case, the INR/dose ratio decreased by 29%–70% during co‐administration of carbamazepine. Additionally, this case demonstrates that the carbamazepine‐induced decrease in the anticoagulant effect of warfarin was concentration‐dependent. An inverse correlation has been reported between the trough concentrations of the CYP3A4 substrate darunavir and carbamazepine, which induce not only CYP2C9 but also CYP3A4 [12]. These findings suggest that concentration‐dependent drug–drug interactions with carbamazepine are likely due to the enhanced induction of CYP2C9 as carbamazepine levels rise. The estimated carbamazepine *C*_min_ ranged from 2.7 to 9.2 μg/mL in this case, suggesting that carbamazepine may reduce the anticoagulant effect of warfarin even at serum levels below the upper limit of the therapeutic range of 4–12 μg/mL. However, predicting changes in PT‐INR based on carbamazepine dose is difficult, as dose escalation leads to enhanced carbamazepine CL.

Information regarding the combined effects of carbamazepine and sodium valproate on the anticoagulant activity of warfarin is lacking. The CYP2C9 inhibitor sodium valproate potentiates the anticoagulant activity on warfarin, in contrast to the attenuating effect of carbamazepine [5]. In the present case, the PT‐INR gradually declined from 2.43 to 1.29 during the warfarin dose of 3.5 mg/day with co‐administration of carbamazepine and sodium valproate, even after dose reduction of carbamazepine and dose escalation of sodium valproate. This result cannot be explained by changes in serum carbamazepine concentrations or CYP2C9 induction or inhibition caused by these two drugs. Although a previous report showed an association between reduced cardiac function and increased PT‐INR, no change in cardiac function was observed before and after the initiation of sodium valproate (BNP, 6.1–31.5 pg/mL; LVEF, 34%–49%) [13]. Therefore, careful monitoring of the anticoagulant effect of warfarin is necessary when it is co‐administered with both carbamazepine and sodium valproate.

In the present case, a 3.8‐fold increase in the PT‐INR was observed following the discontinuation of carbamazepine. This finding was similar to the previous reports indicating that discontinuation of carbamazepine can cause a 1.6‐fold increase in PT‐INR and a 5‐fold increase in prothrombin time [11, 14]. The elevation of PT‐INR in our case may be more pronounced due to the co‐administration of sodium valproate and a slight dose escalation of warfarin from 3.5 to 3.8 mg/day. These findings indicate that the warfarin dosage should be carefully adjusted when discontinuing carbamazepine, regardless of the co‐administration of sodium valproate.

## 4 Conclusion

This case highlights an inverse correlation between serum carbamazepine concentration and INR/dose ratio in anticoagulant therapy with warfarin. However, this association was not observed during tapering of the carbamazepine dose after co‐administration with sodium valproate. Careful monitoring of the anticoagulant effects of warfarin is essential before discontinuing carbamazepine.

## Author Contributions

**Yuki Shirayama:** conceptualization, data curation, formal analysis, investigation, methodology, project administration, visualization, writing – original draft. **Yu Takasao:** conceptualization, data curation, formal analysis, investigation. **Toshiyuki Shinozaki:** investigation, writing – review and editing. **Masayuki Abiko:** investigation, writing – review and editing. **Kota Suzuki:** investigation, writing – review and editing. **Kosuke Doki:** data curation, formal analysis, methodology, writing – review and editing.

## Funding

This study received no specific grants from any funding agency in the public, commercial, or not‐for‐profit sectors.

## Ethics Statement

This study was approved by the Human Research Ethics Committee of Tsuruha Holdings (approval number: HD2025011; recognition date: April 4, 2024).

## Consent

Written informed consent was obtained from the patient for publication of this report.

## Conflicts of Interest

The authors declare no conflicts of interest.

## Data Availability

The data that support the findings of this study are available from the corresponding author upon reasonable request.
